# Differential utilization of ketone bodies by neurons and glioma cell lines: a rationale for ketogenic diet as experimental glioma therapy

**DOI:** 10.1186/1471-2407-11-315

**Published:** 2011-07-26

**Authors:** Gabriele D Maurer, Daniel P Brucker, Oliver Bähr, Patrick N Harter, Elke Hattingen, Stefan Walenta, Wolfgang Mueller-Klieser, Joachim P Steinbach, Johannes Rieger

**Affiliations:** 1 Dr. Senckenberg Institute of Neurooncology, Goethe University Hospital, Schleusenweg 2-16, 60528 Frankfurt, Germany; 2 Institute of Neurology (Edinger Institute), Goethe University Hospital, Heinrich-Hoffmann-Str. 7, 60528 Frankfurt, Germany; 3 Institute of Neuroradiology, Goethe University Hospital, Schleusenweg 2-16, 60528 Frankfurt, Germany; 4 Institute of Physiology and Pathophysiology, Gutenberg University Medical Center, Duesbergweg 6, 55099 Mainz, Germany

## Abstract

**Background:**

Even in the presence of oxygen, malignant cells often highly depend on glycolysis for energy generation, a phenomenon known as the Warburg effect. One strategy targeting this metabolic phenotype is glucose restriction by administration of a high-fat, low-carbohydrate (ketogenic) diet. Under these conditions, ketone bodies are generated serving as an important energy source at least for non-transformed cells.

**Methods:**

To investigate whether a ketogenic diet might selectively impair energy metabolism in tumor cells, we characterized *in vitro *effects of the principle ketone body 3-hydroxybutyrate in rat hippocampal neurons and five glioma cell lines. *In vivo*, a non-calorie-restricted ketogenic diet was examined in an orthotopic xenograft glioma mouse model.

**Results:**

The ketone body metabolizing enzymes 3-hydroxybutyrate dehydrogenase 1 and 2 (BDH1 and 2), 3-oxoacid-CoA transferase 1 (OXCT1) and acetyl-CoA acetyltransferase 1 (ACAT1) were expressed at the mRNA and protein level in all glioma cell lines. However, no activation of the hypoxia-inducible factor-1α (HIF-1α) pathway was observed in glioma cells, consistent with the absence of substantial 3-hydroxybutyrate metabolism and subsequent accumulation of succinate. Further, 3-hydroxybutyrate rescued hippocampal neurons from glucose withdrawal-induced cell death but did not protect glioma cell lines. In hypoxia, mRNA expression of OXCT1, ACAT1, BDH1 and 2 was downregulated. *In vivo*, the ketogenic diet led to a robust increase of blood 3-hydroxybutyrate, but did not alter blood glucose levels or improve survival.

**Conclusion:**

In summary, glioma cells are incapable of compensating for glucose restriction by metabolizing ketone bodies *in vitro*, suggesting a potential disadvantage of tumor cells compared to normal cells under a carbohydrate-restricted ketogenic diet. Further investigations are necessary to identify co-treatment modalities, e.g. glycolysis inhibitors or antiangiogenic agents that efficiently target non-oxidative pathways.

## Background

High-grade gliomas are intrinsic brain tumors characterized by resistance to apoptotic stimuli, diffuse infiltration into the surrounding tissue and local immunosuppression. Despite advances in research on tumor biology and efforts to promote new therapies, the prognosis for patients with high-grade gliomas is still poor. Currently available treatment options for glioblastoma patients, including surgery, radio- and chemotherapy, result in a median survival of only about 12 months [1,2]. Obviously, other therapeutic approaches are needed that, on the one hand, impair tumor cell growth and, on the other hand, permit an adequate quality of life.

**Figure 1:**
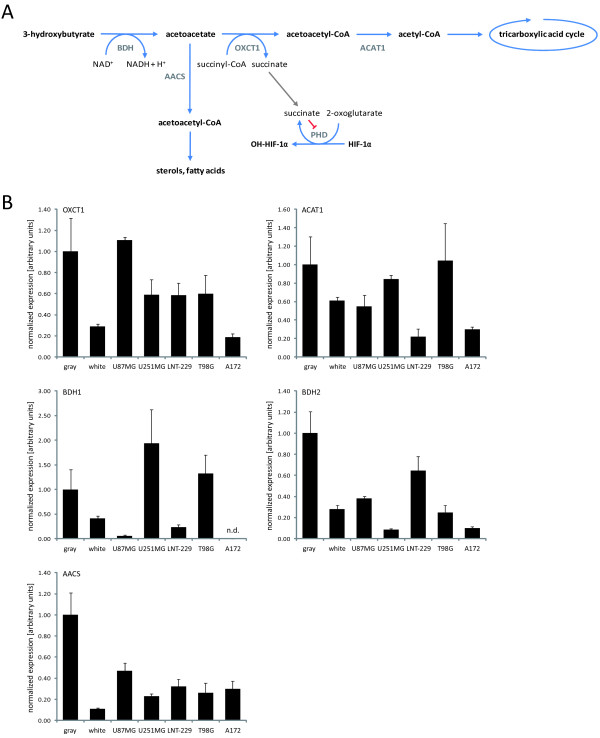
**(A) Simplified diagram of cerebral ketone body metabolism**. At times of glucose shortage, such as prolonged fasting, ketone bodies are an important energy source for the brain. The oxidoreductase 3-hydroxybutyrate dehydrogenase (BDH) mediates the first step of ketone body degradation, between 3-hydroxybutyrate and acetoacetate. 3-oxoacid-CoA transferase 1 (OXCT1) catalyzes the transfer of coenzyme A from succinyl-CoA to acetoacetate, generating acetoacetyl-CoA. Via acetyl-CoA acetyltransferase 1 (ACAT1), acetoacetyl-CoA is converted into two molecules of acetyl-CoA, which then enter the citric acid cycle. The utilization of ketone bodies results in an elevation of intracellular succinate, leading to HIF-1α stabilization via product inhibition of prolyl hydroxylases (PHD). Furthermore, ketone bodies provide substrates for the synthesis of various molecules, especially lipids. In this regard, acetoacetyl-CoA is formed in the cytoplasm from acetoacetate by the action of acetoacetyl-CoA synthetase (AACS). **(B) **The expression of the ketone body metabolizing enzymes was analyzed in five glioma cell lines as well as in normal brain (gray, gray matter; white, white matter) by real-time quantitative PCR. Results are presented as the fold change in gene expression normalized to the internal control 18S rRNA (mean and standard deviation; n.d., not detectable).

Many malignant cells display high rates of glycolysis and lactate production, even in the presence of adequate oxygen, a phenomenon known as aerobic glycolysis or the Warburg effect [3]. Additionally, tumor hypoxia results in constitutive upregulation of glycolysis and is considered to substantially contribute to the resistance of tumor cells to therapeutic strategies [4,5]. One possibility to affect the metabolism of such "glucose dependent" tumors could be the ketogenic diet. The classic ketogenic diet is a high-fat and low-carbohydrate dietetic approach raising levels of serum ketone bodies, i.e. acetoacetate, 3-hydroxybutyrate (from less than 0.1 mM to 0.2-1.8 mM and 2-5 mM, respectively) and acetone, and lowering brain glucose uptake [6-8]. Acetoacetate and 3-hydroxybutyrate are almost exclusively synthesized in the liver from acetyl-CoA that results from the beta-oxidation of fatty acids. Acetone is formed from acetoacetate by spontaneous decarboxylation and is generally considered of little metabolic significance. Energy generation from ketone bodies takes place via the citric acid cycle and oxidative phosphorylation and therefore requires proper mitochondrial function. In contrast to glucose, ketone bodies thus bypass cytoplasmic glycolysis and directly enter the citric acid cycle as acetyl-CoA. In fasting humans, the water-soluble ketone bodies can supply approaching 60% of the brain's energy requirement. Besides being a source of energy, ketone bodies can provide substrates for anabolism, particularly for the synthesis of lipids such as cholesterol in myelin. A simplified diagram of ketone body metabolism is shown in Figure 1A. As malignant cells are thought to depend on glucose as a major source of energy while having impaired mitochondrial function [9-11], a ketogenic diet thus might induce a tumor-selective energy deprivation. There has been considerable research on the safety, the anticonvulsant and neuroprotective effects of the ketogenic diet [8,12,13], whereas less attention has been paid to its potential for tumor therapy. Similar to tumors of peripheral tissues [14], glial tumors were found to have less activity of 3-oxoacid-CoA transferase 1 (OXCT1), the rate-limiting enzyme of ketone body degradation, than normal human white (and gray) matter [15]. Following encouraging observations in two female pediatric patients with advanced stage malignant astrocytoma who seemed to benefit from a ketogenic diet [16], a reduction of glioma growth was noticed in orthotopic mouse models when a calorie-restricted ketogenic diet was applied [17,18]. A first clinical study assessing the feasibility and safety of a non-calorie-restricted ketogenic diet in patients with recurrent glioblastoma (the ERGO trial, NCT00575146) revealed good tolerability and suggested some antitumor activity [19]. Nevertheless, to date, little data concerning a potential application of a ketogenic diet in brain tumor treatment is available. So far no randomized controlled trials have been initiated on the ketogenic diet for tumor therapy. Further, biological effects and associated metabolic alterations of a ketogenic diet on glioma versus neural cells remain to be elucidated. In the present study, we therefore characterized *in vitro *effects primarily of 3-hydroxybutyrate, the major circulating ketone body, on five human glioma cell lines (U87MG, U251MG, LNT-229, T98G and A172) and rat hippocampal neurons and examined the efficacy of a non-calorie-restricted ketogenic diet in an orthotopic glioma xenograft mouse model.

## Methods

### Reagents

Acetoacetate (lithium salt), 3-hydroxybutyrate (sodium salt), 3-nitropropionic acid, 3-(4,5-dimethyl-2-thiazolyl)-2,5-diphenyl-2H-tetrazolium bromide (MTT), rotenone, 3-bromopyruvate and PCR primers were purchased from Sigma-Aldrich (St. Louis, MO), PolyFect (used for transfection of A172 cells) and Attractene (used for transfection of U87MG, U251MG, LNT-229 and T98G cells) transfection reagents from Qiagen (Hilden, Germany), recombinant human TRAIL from PeproTech (Rocky Hill, NJ), temozolomide from Axxora (San Diego, CA). Antibodies used were anti-ACAT1 (Sigma-Aldrich), anti-actin (sc1616, Santa Cruz Biotechnology), anti-BDH1 (Sigma-Aldrich), anti-BDH2 (Sigma-Aldrich), anti-HIF-1α (BD Transduction Laboratories, San Jose, CA) and anti-OXCT1 (ProteinTech, Chicago, IL). The 3HRE-pTK-luc reporter construct was a kind gift from J. Pouysségur [20-22]. Acetoacetate is an unstable compound and commercially available only as a lithium salt. Lithium itself is known to have pleiotropic effects on diverse cell processes. By contrast, 3-hydroxybutyrate, the major ketone body in blood, is chemically stable and provided as a sodium salt. We therefore concentrated on 3-hydroxybutyrate in most of our experiments.

### Cell culture

The human malignant glioma cell lines T98G and U87MG and NIH-3T3 murine fibroblast cells were obtained from the American Type Culture Collection (Manassas, VA). A172, LNT-229 and U251MG cells were kindly provided by N. de Tribolet (Lausanne, Switzerland). LNT-229 cells expressing a short-hairpin construct for p53 gene suppression (LNT-229 p53sh) and the corresponding control cells (LNT-229 scrambled sh) have been described [23]. Glioma cells were maintained at 37°C and 5% CO_2 _in Dulbecco's modified Eagle's Medium (4500 mg/L glucose; Sigma-Aldrich) with 10% fetal calf serum (PAA, Pasching, Austria), 2 mM glutamine, 100 IU/mL penicillin and 100 μg/mL streptomycin. Primary hippocampal neurons and astrocytes from newborn Wistar rats were prepared as described [24,25]. Unless otherwise indicated, experiments were performed in serum-free medium containing 5 mM glucose (control), supplemented with 5 mM acetoacetate (lithium chloride in the corresponding control) or 5 mM 3-hydroxybutyrate. For some experiments, glucose was added to serum- and glucose-free medium to give final concentrations of 0, 1, 2.5, 5, 10 and 25 mM. For hypoxic conditions, cells were cultured in a Binder CB53 incubator (Binder, Tuttlingen, Germany). With institutional review board approval (University Cancer Center Frankfurt), specimens of normal human gray and white matter were collected and stored at -80°C until RNA extraction.

### Growth and viability assays

Cell density was assessed using crystal violet staining, resolubilizing the dye in 0.1 M sodium citrate and measuring the absorbance at 560 nm (Multiskan™ EX, Thermo Scientific, Langenselbold, Germany). In some experiments, cell viability was analyzed by MTT reduction assay [26]; formazan crystals were dissolved in dimethyl sulfoxide and the absorbance was read at 595 nm. For the evaluation of cell proliferation, incorporation of bromodeoxyuridine (BrdU) was determined according to the manufacturer's instructions (BrdU Cell Proliferation ELISA, Roche Diagnostics, Mannheim, Germany). Clonogenic survival assays were performed by seeding 500 cells in 6-well plates and exposing them to temozolomide for 24 h, followed by further observation in drug-free medium containing 5 mM glucose, 5% fetal calf serum (control) and 5 mM 3-hydroxybutyrate. After crystal violet staining, colonies of more than 50 cells were counted [27].

### Determination of glucose, lactate and 3-hydroxybutyrate

Glucose and lactate concentrations of cell-free supernatants were measured on a Hitachi 917 analyzer (Roche Diagnostics). For the assessment of 3-hydroxybutyrate levels, a Precision Xtra^R ^monitoring system (Abbott Laboratories, Abbott Park, IL) was used.

### SDS-PAGE and immunoblotting

For the preparation of protein extracts, cells were harvested and lysed in a buffer containing 50 mM Tris-HCl, 120 mM NaCl, 5 mM EDTA, 0.5% Nonidet-P40, 2 μg/mL aprotinin, 10 μg/mL leupeptin, 100 μg/mL phenylmethylsulfonyl fluoride, 50 mM NaF, 200 μM NaVO_5 _and phosphatase inhibitor cocktails I and II (Sigma-Aldrich). Protein concentrations were determined using a Bradford assay (Bio-Rad, Hercules, CA). Equal amounts of total protein were fractionated under reducing conditions by sodium dodecyl sulfate polyacrylamide gel electrophoresis (SDS-PAGE) and electroblotted on nitrocellulose (Amersham, Braunschweig, Germany). Membranes were blocked in Tris buffered saline containing 5% skim milk and 0.1% Tween-20 and incubated with the appropriate primary and secondary antibodies. Immune complexes were detected by enhanced chemiluminescence (Pierce, Rockford, IL).

### Real-time quantitative PCR

Total RNA was extracted using TRIzol™ (Invitrogen, Carlsbad, CA) and the RNeasy™ system (Qiagen), cDNA was generated with SuperScript VILO™ (Invitrogen). Real-time PCR was performed in triplicate reactions using ABsolute™ Blue QPCR SYBR Green Fluorescein Mix (Thermo Fisher Scientific, Waltham, MA) and the iQ5 real-time PCR detection system (BioRad, Munich, Germany). Gene expression was calculated relative to the internal control 18S ribosomal RNA (iQ5 software, BioRad). Human-specific primer sequences: 18S rRNA, forward 5'-CGGCTACCACATCCAAGGAA-3', reverse 5'-GCTGGAATTACCGCGGCT-3', AACS, forward 5'-ACTGCAGAATCAACCCCAAG-3', reverse 5'-TTGCCGTTGAGCGTATACAA-3', ACAT1, forward 5'-GGAGAGCATGTCCAATGTTCC-3', reverse 5'-CGTCCTGTTCATTTCGTGCAA-3', BDH1, forward 5'-TGGTTTTGGAACCACCGGGAGGA-3', reverse 5'-GCTCCGCCGCACTGGCATAA-3', BDH2, forward 5'-GGCCGCTGCTCAGGGGATTG-3', reverse 5'-ACGGCTGCCTTGGTTGTGCT-3', GLUT1, forward 5'-GATTGGCTCCTTCTCTGTGG-3', reverse 5'-TCAAAGGACTTGCCCAGTTT-3', MCT4, forward 5'-ATTGGCCTGGTGCTGCTGATG-3', reverse 5'-CGAGTCTGCAGGAGGCTTGTG-3' [28], OXCT1, forward 5'-CACCAGTGCTCATCGCCATA-3', reverse 5'-CACATAGCCCAAAACCACCAA-3', VEGF, forward 5'-CTACCTCCACCATGCCAAGT-3', reverse 5'-ATGTTGGACTCCTCAGTGGG-3'. Rat-specific primer sequences: 18S rRNA, forward 5'-GTTGGTTTTCGGAACTGAGGC-3', reverse 5'-GTCGGCATCGTTTATGGTCG-3', AACS, forward 5'-ACCGGCTCGCCACTGAAAGC-3', reverse 5'-ATGGAGCCGAGGAGCACGGT-3', ACAT1, forward 5'-GGGCTTCCGCCGTGCTGATT-3', reverse 5'-CAGCGGGTCACGTGGAACTGT-3', BDH1, forward 5'-GTCAGACGAGCGCACCGGTC-3', reverse 5'-GGCCAGCATCATGGCACCGA-3', BDH2, forward 5'-AGGTCGCCCTGCTCTGCGTA-3', reverse 5'-GCTCACCCGGCCAGTTTGCT-3', OXCT1, forward 5'-AGCCCGGAGAAGACGTCAGGG-3', reverse 5'-ATGCGCATTCCCCTTTGCGGAG-3'.

### Luciferase reporter assay

Cells were transiently transfected with a 3HRE-pTK-luc firefly and, for normalization of transfection efficiency, a pRL-CMV renilla luciferase construct. Luciferase activity was assayed using a dual luciferase reporter assay system [29] and an Infinite^R ^M200 PRO microplate reader (Tecan, Maennedorf, Switzerland).

### Invasion and migration assays

Matrigel invasion assays were performed as described previously with some modifications [30]. Transwell chambers (12 mm diameter, 8 μm pore size, Corning Costar, Acton, MA) were precoated with 10 μg/cm^2^ Matrigel (Matrigel™ Basement Membrane Matrix, BD Biosciences, Bedford, MA); NIH-3T3-conditioned medium was used as a chemoattractant. Following 12 h incubation, migrated or invaded cells were fixed, stained and counted by microscopic examination.

### Animals and diets

All animal work was performed in accordance with the National Institutes of Health guidelines *Guide for the Care and Use of Laboratory Animals *and institutional standards. 24 female 8-10 week-old athymic mice (Foxn1nu, Harlan, Indianapolis, IN) were maintained in groups of 6 animals per cage in a pathogen-free environment and given *ad libitum *access to food and water. On day 0 of the experiment, 10^5 ^human LNT-229 glioma cells were stereotactically implanted into the right striatum. Following recovery from surgery (day 1), animals were randomized into two diet groups, a standard diet rich in carbohydrates versus a ketogenic diet. The standard diet was provided by the animal feed manufacturer ssniff Spezialdiaeten GmbH (Soest, Germany), the ketogenic diet was kindly supplied by J.F. Coy (Tavarlin AG, Darmstadt, Germany; [31]). Diet characteristics are summarized in Table 1. Animal body weight was measured twice weekly. Neurological symptoms were assessed daily by modified neurological scores (grade 0: normal; grade 1: tail weakness or tail paralysis; grade 2: hind leg paraparesis or hemiparesis; grade 3: hind leg paralysis or hemiparalysis; grade 4: complete paralysis (tetraplegia), moribund stage or death). Animals were killed at the onset of neurological symptoms equal or worse than grade 2.

**Table 1:** Composition of the standard and ketogenic diets

Component	Control, standard diet	Ketogenic diet
**Fat**	6.1	35.5
**Carbohydrate**	55.6	0.2
**Protein**	21.8	13.0
**Fiber**	3.8	14.8
**Ashes**	5.3	2.1
**Energy [kJ/g]**	15.8	15.4
**Ketogenic ratio**	0.08:1	2.7:1

Components of the diets used are listed in grams per 100 grams of food. The fat in the standard diet derived from soybean oil; the fat source of the ketogenic diet consisted of a mixture of vegetable oils from flaxseed and hempseed with elevated levels of omega-3 fatty acid and medium-chain triglycerides [31]. The ketogenic ratio was calculated according to the following formula: Fats/(Protein + Carbohydrates).

### Magnetic resonance imaging (MRI)

Imaging was performed in prone position on days 37 and 65 after tumor cell implantation using a 3-Tesla MRI scanner (Trio^R^, Siemens, Erlangen, Germany), a circular polarized wrist coil and 0.5 mmol/mL gadolinium-diethylenetriaminepentaacetic acid (Magnevist^R^, Bayer Schering Pharma, Berlin, Germany). After intraperitoneal injection of 0.3 mL/animal, standard T2-weighted and T1-weighted sequences were acquired.

### Determination of blood glucose, blood 3-hydroxybutyrate and serum IGF-1

Blood glucose and 3-hydroxybutyrate levels were measured on the day of tumor cell implantation and every 7 days thereafter using 2 μL of peripheral blood from the tail vein and a Precision Xtra^R ^monitoring system. Mouse serum IGF-1 concentrations were analyzed by immunoassay (Quantikine^R^, R&D Systems, Minneapolis, MN).

### Histology/Immunohistochemistry

Mouse brains were formalin-fixed and paraffin-embedded. 4 μm thick sections were cut and deparaffination procedures were performed according to standard protocols. Hematoxylin and eosin stainings were analyzed by an experienced investigator (PNH). Immunohistochemistry was carried out using a monoclonal mouse antibody against human Ki67-antigen (clone MIB-1, Dako, Glostrup, Denmark) and the Ventana Discovery IHC System (Ventana, Strasbourg, France). Nuclear staining was scored as positive. Five randomly picked fields (3.7 mm^2^) per specimen were evaluated.

### Metabolic mapping using bioluminescence imaging

The technique of *bioluminescence imaging *allows for the spatial and quantitative detection of key metabolites of energy metabolism in cryosections of human or animal tissues [32,33]. Briefly, heat-inactivated cryostat sections prepared from rapidly frozen brains (three animals per diet group) were immersed into an enzyme solution using a temperature-controlled chamber placed on a microscope stage. The solution contains enzymes that link the metabolite of interest, i.e. ATP, glucose or lactate, to bacterial or firefly luciferase. Subsequently, the induced light emission was registered by a photon detecting video system (Andor EMCCD DU888, BFI-Optilas, Munich, Germany) connected to the microscope (Axiophot, Zeiss, Oberkochen, Germany). The registered intensity values are proportional to the local metabolite concentrations. Therefore, the resulting digital images could be calibrated [μmol/g] using appropriate tissue standards that were processed in the same way as the brain sections. Such distributions could be displayed routinely as color-coded images. Average concentration values were acquired in designated tumor regions and normal tissue using *digital overlay *of the metabolite distributions with images from parallel cryosections stained with hematoxylin and eosin.

### Statistical Analysis

*In vitro *experiments were performed at least three times with similar results. Data analysis was carried out with SPSS version 17.0 (IBM SPSS, Chicago, IL). Significance was tested using the two-tailed Student's t-test. Synergy was assessed by the fractional product method [34]. Survival was estimated by Kaplan-Meier analysis, and differences were tested by Mantel-Cox log-rank statistics.

## Results

*Ketone body metabolizing enzymes are expressed in the five glioma cell lines both at the mRNA and the protein level*. First, we examined whether the five glioma cell lines express key enzymes involved in ketone body metabolism. U87MG, U251MG, LNT-229, T98G and A172 cells exhibited expression of 3-hydroxybutyrate dehydrogenase (BDH), 3-oxoacid-CoA transferase (OXCT1), acetyl-CoA acetyltransferase (ACAT1) and acetoacetyl-CoA synthetase (AACS) at the mRNA (Figure 1B) and the protein level (Figure 3B). No consistent change in expression was observed following exposure to 3-hydroxybutyrate for 24 h or 48 h (Figure 3B and data not shown). In rat hippocampal neurons, the expression of these enzymes was confirmed by real-time quantitative PCR (data not shown).

**Figure 2:**
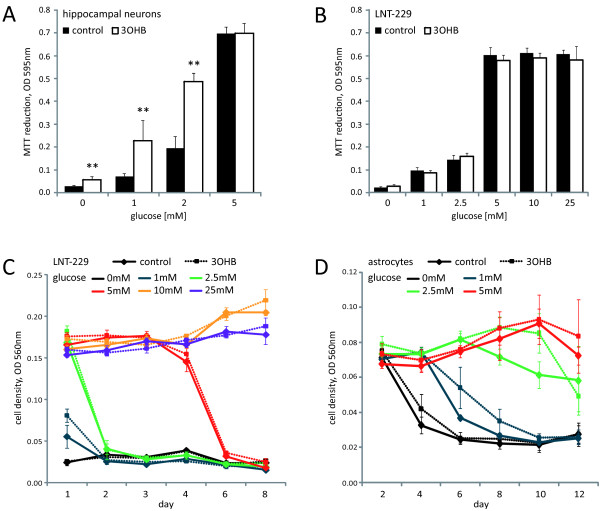
**3-hydroxybutyrate protects neonatal rat hippocampal neurons from cell death induced by glucose deprivation**. Neonatal rat hippocampal neurons **(A) **or LNT-229 glioma cells **(B) **were cultured at different glucose concentrations in the absence (control) or presence of 3-hydroxybutyrate (3OHB, 5 mM). MTT reduction was determined 120 h (hippocampal neurons) or 72 h (LNT-229) after exposure (mean and standard deviation, ** p < 0.01). **(C) **Glioma cells were grown in medium containing 0 mM, 1 mM, 2.5 mM, 5 mM, 10 mM or 25 mM glucose, supplemented with 3-hydroxybutyrate (3OHB, 5 mM) or not. Cell density was assessed by crystal violet staining at day 1, 2, 3, 4, 6 and 8 after exposure, as shown here for LNT-229 cells (mean and standard deviation). **(D) **Primary rat astrocytes were treated similarly and crystal violet staining was performed at day 2, 4, 6, 8, 10 and 12 (mean and standard deviation). For a clearer arrangement, 10 mM and 25 mM glucose conditions, showing no difference in cell density between control and 3-hydroxybutyrate supplementation, are not displayed.

*3-hydroxybutyrate protects primary rat hippocampal neurons but not human glioma cell lines against glucose deprivation-induced cell death*. Having confirmed the expression of ketone body metabolizing enzymes, we assessed whether 3-hydroxybutyrate could serve as an alternative energy source under conditions of reduced glucose availability. For this purpose, rat hippocampal neurons or glioma cells were incubated in the absence or presence of 5 mM 3-hydroxybutyrate in serum-free medium containing different glucose concentrations (hippocampal neurons: 0 mM, 1 mM, 2 mM or 5 mM; glioma cell lines: 0 mM, 1 mM, 2.5 mM, 5 mM, 10 mM or 25 mM), and cell density was analyzed by MTT assay (Figure 2A-B) or crystal violet staining (Figure 2C). Primary rat hippocampal neurons benefited from the presence of 3-hydroxybutyrate by prolonged survival under glucose deprivation. By contrast, in no glioma cell line, a rescue from glucose withdrawal-induced cell death by this ketone body was detectable. In untransformed primary rat astrocytes, some advantage of 3-hydroxybutyrate supplementation was evident, although to a lesser extent than in primary rat hippocampal neurons (Figure 2D).

**Figure 3:**
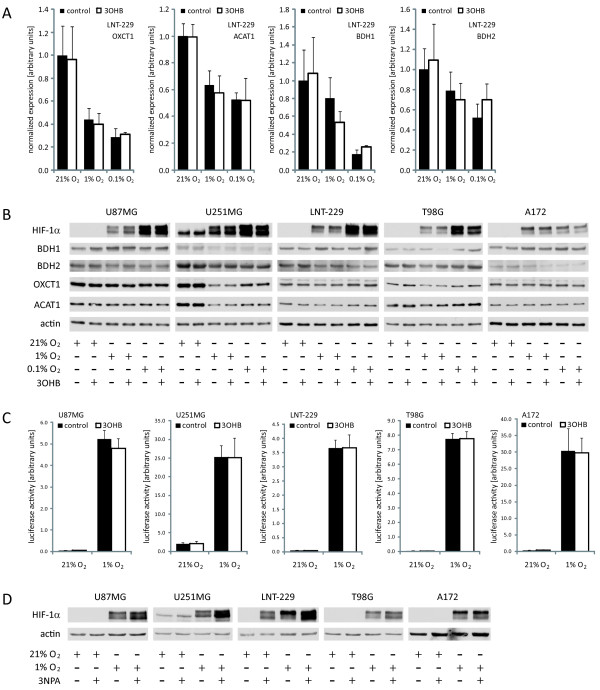
**Influence of hypoxia and 3-hydroxybutyrate on the expression of ketone body metabolizing enzymes and the activity of HIF-1α signaling**. **(A) **LNT-229 glioma cells were cultured in the absence or presence of 3-hydroxybutyrate (3OHB, 5 mM) at 21%, 1% or 0.1% oxygen for 24 h. mRNA levels of OXCT1, ACAT1, BDH1 and BDH2 were determined by real-time quantitative PCR. Data are presented as the fold change in gene expression normalized to the internal control 18S rRNA (mean and standard deviation, p < 0.05 compared with normoxic conditions, asterisks omitted for clarity). **(B) **Glioma cell lines were treated as in **(A)**, and the expression of HIF-1α, BDH1, BDH2, OXCT1 and ACAT1 was analyzed by immunoblot. **(C) **HIF-specific transcriptional activity was examined by luciferase reporter assay (3HRE-pTK-luc construct) in the absence or presence of 3-hydroxybutyrate (24 h treatment, mean and standard deviation). **(D) **Glioma cells were incubated for 24 h at the indicated oxygen conditions in the absence or presence of 3-nitropropionic acid (3NPA, 10 mM), and the expression of HIF-1α was analyzed by immunoblot.

*Ketone bodies do not alter growth or proliferation of glioma cell lines*. Next, we analyzed potential effects of ketone bodies on cell growth and proliferation in the presence of sufficient glucose. In all glioma cell lines tested, the presence of ketone bodies, i.e. acetoacetate, 3-hydroxybutyrate, or both, did not alter cell density at different time points up to 84 h after exposure (Table 2 and data not shown). Furthermore, cell proliferation as assessed by BrdU-uptake was unaffected by 3-hydroxybutyrate (data not shown). As glucose availability has been demonstrated to be relevant for cellular survival under hypoxic conditions [35-37], we investigated whether hypoxia might alter the deficiency of glioma cells to metabolize ketone bodies. However, at different oxygen (21%, 1% or 0.1% O_2_) or glucose concentrations, offering 3-hydroxybutyrate did not influence cell density (Table 2 and data not shown). Furthermore, the presence of 3-hydroxybutyrate did not modify glucose consumption or lactate generation of the glioma cell lines examined (analyzed up to six days after exposure; Additional file 1, Figure S1). Because 3-hydroxybutyrate might be metabolized for cellular processes which would not directly be reflected by differences in glucose consumption, 3-hydroxybutyrate levels were measured in cell culture supernatants. Only a marginal decrease in 3-hydroxybutyrate in the glioma cell culture supernatants was observed (between 0.0 mM and 0.5 mM; analyzed for up to six days after exposure), indicating no substantial utilization of this additional energy source. In cultures of rat hippocampal neurons, 3-hydroxybutyrate supernatant concentrations were reduced to a greater extent despite lower cell densities (1.3 mM decrease after three days of exposure). A loss of p53 function has been shown to result in metabolic alterations such as reduced cellular respiration, increased glucose consumption and lactate production [38]. Therefore, we examined whether antagonizing p53 would modulate the metabolism of 3-hydroxybutyrate. However, cell density of both p53 wild-type LNT-229 cells and of LNT-229 cells depleted of p53 by stable shRNA expression (LNT-229 p53sh) did not differ in the absence or presence of 3-hydroxybutyrate (Table 2 and data not shown), indicating no induction of ketone body metabolism by inhibition of p53 function.

**Table 2:** Growth curves in glioma cells exposed to vehicle or ketone bodies

21% O_2_	3OHB	AcAc	LiCl	3OHB + AcAc	3OHB + LiCl
**U87MG**	94.9 ± 9.9	109.7 ± 10.3	104.5 ± 11.7	107.3 ± 10.1	100.8 ± 10.0
**U251MG**	100.2 ± 5.8	97.4 ± 5.5	97.0 ± 6.9	96.1 ± 5.4	90.2 ± 5.0
**LNT-229**	98.4 ± 4.7	100.6 ± 3.7	109.5 ± 7.9	104.0 ± 3.6	95.7 ± 4.0
**T98G**	96.3 ± 4.8	87.6 ± 4.0	91.2 ± 7.8	86.1 ± 5.3	88.0 ± 4.1
**A172**	97.1 ± 6.0	100.3 ± 5.7	99.9 ± 7.4	84.9 ± 10.7	90.7 ± 5.6
**LNT-229 scrambled sh**	99.9 ± 9.5	109.2 ± 6.0	103.7 ± 5.3	102.2 ± 6.2	97.5 ± 6.8
**LNT-229 p53sh**	98.3 ± 5.3	102.3 ± 3.2	96.5 ± 2.4	95.1 ± 2.4	95.0 ± 2.8

**0.1% O_2_**	**3OHB**	**AcAc**	**LiCl**	**3OHB + AcAc**	**3OHB + LiCl**

**U87MG**	97.7 ± 4.9	115.8 ± 5.8	115.2 ± 4.9	108.8 ± 4.2	104.0 ± 5.7
**U251MG**	96.6 ± 2.7	102.6 ± 5.0	98.1 ± 2.5	92.1 ± 5.4	86.9 ± 3.0
**LNT-229**	93.6 ± 3.5	103.2 ± 4.9	104.4 ± 3.3	97.0 ± 3.3	91.0 ± 5.1
**T98G**	98.8 ± 10.1	100.2 ± 9.1	97.3 ± 8.7	101.7 ± 7.3	91.5 ± 9.9
**A172**	107.9 ± 15.6	93.2 ± 9.2	95.8 ± 7.4	101.0 ± 8.7	91.6 ± 6.8
**LNT-229 scrambled sh**	99.6 ± 5.4	104.5 ± 4.7	105.4 ± 6.0	98.9 ± 4.6	103.9 ± 8.2
**LNT-229 p53sh**	94.8 ± 2.5	107.4 ± 4.2	107.6 ± 5.4	102.9 ± 4.0	96.0 ± 4.5

Cells were exposed to 3-hydroxybutyrate (3OHB, 5 mM), acetoacetate (AcAc, 5 mM), lithium chloride (LiCl, 5 mM) or both for 36 h at 21% or 0.1% O_2 _and subsequently stained with crystal violet. Data are expressed as percentages (mean and standard deviation) relative to untreated control cells.

*Exposure to 3-hydroxybutyrate does not modulate glioma cell motility and invasiveness*. The infiltrative behavior of malignant glioma cells is a function of two phenotypes, migration and invasiveness. Migration refers to the capacity of locomotion whereas invasion involves migration plus a degradative function achieved by the liberation of proteolytic enzymes. Using classical migration and matrigel invasion assays, we did not detect any significant changes when 3-hydroxybutyrate was offered (Table 3).

**Table 3:** Effects of 3-hydroxybutyrate on migratory and invasive abilities

	U87MG	U251MG	LNT-229	T98G	A172
**migration**	94.3 ± 7.8	80.7 ± 31.2	98.4 ± 18.6	100.8 ± 5.7	95.6 ± 10.0
**invasiveness**	121.4 ± 17.0	104.0 ± 18.6	95.3 ± 14.3	111.4 ± 21.8	108.4 ± 9.2

U87MG, U251MG, LNT-229, T98G or A172 cells were exposed to 3-hydroxybutyrate (5 mM) and migration or invasiveness were assessed. Data are expressed as mean percentages relative to control cultures and standard error of the mean.

*Treatment with 3-hydroxybutyrate does not modify the expression of hypoxia-inducible factor-1α (HIF-1α) and of its target genes*. Human and murine solid tumors are characterized by hypoxic areas with intratumoral pO_2 _values between 2 and 12 mmHg (approximately 0.3% - 2% O_2_; [39-41]). We therefore looked at the expression of ketone body metabolizing enzymes under hypoxia (1% or 0.1% O_2_) as well. Regardless of the absence or presence of 3-hydroxybutyrate, we noticed a considerable downregulation of BDH, OXCT1 and ACAT1 mRNA levels in all cell lines (Figure 3A and data not shown) in hypoxia. At 24 h after 3-hydroxybutyrate exposure, this downregulation was visible on protein level primarily in U251MG cells (Figure 3B). Succinate is generated during the activation of acetoacetate to acetoacetyl-CoA by the enzyme OXCT1. Accumulation of succinate can result in the stabilization of HIF-1α via product inhibition of prolyl hydroxylase (PHD) enzymes [42]. The small amounts of 3-hydroxybutyrate which might be metabolized by glioma cells (between 0.0 mM and 0.5 mM decrease in supernatant level; see above) could modulate HIF-1α expression as an indirect hint for ketone body degradation. However, in these cell lines, 3-hydroxybutyrate did not induce an elevation of HIF-1α protein (Figure 3B). Similarly, we did not observe a rise in HRE reporter gene activity (Figure 3C) or in expression of the HIF-1α target genes *GLUT1, VEGF *and *MCT4 *(Additional file 2, Figure S2, and data not shown) in any of the five glioma cell lines examined. 3-nitropropionic acid (3NPA) is an irreversible inhibitor of succinate dehydrogenase, leading to elevated succinate levels. As shown in Figure 3D, 3NPA led to an increase in HIF-1α protein, at least under hypoxic conditions, confirming that HIF-1α is indeed regulated by succinate. In summary, these results suggest that 3-hydroxybutyrate does not modulate HIF-1α expression or activity in the glioma cell lines analyzed, and therefore provide additional evidence for a defective ketone body utilization of glioma cells.

**Figure 4:**
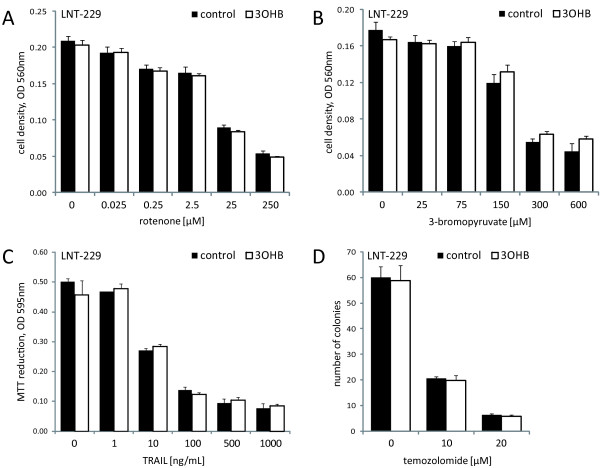
**The presence of 3-hydroxybutyrate does not modify the sensitivity of LNT-229 cells to inhibitors of oxidative phosphorylation, glycolysis, TRAIL or temozolomide**. LNT-229 cells (mean and standard deviation) were treated with increasing concentrations of **(A) **rotenone (48 h), **(B) **3-bromopyruvate (150 min) or **(C) **TRAIL (24 h). In the 3-bromopyruvate experiments, cells were preincubated in medium containing 3-hydroxybutyrate (3OHB, 5 mM) or not. Cell density was evaluated by crystal violet staining **(A, B) **or MTT reduction **(C)**. **(D) **LNT-229 cells were exposed to temozolomide for 24 h, followed by further observation in drug-free medium supplemented with 3-hydroxybutyrate (3OHB, 5 mM) or not, and clonogenic survival was analyzed (mean and standard deviation).

*3-hydroxybutyrate does not influence glioma cell sensitivity towards different triggers of cell death*. Finally, we were interested in whether ketosis could change the susceptibility to different mechanisms of cell death. In U87MG, U251MG, LNT-229, T98G and A172 cells, sensitivity to treatment with rotenone, an inhibitor of mitochondrial respiratory chain complex I, or 3-bromopyruvate, a synthetic brominated derivative of pyruvic acid inhibiting the enzyme hexokinase II, was not altered in the presence of 3-hydroxybutyrate (Figure 4A-B and data not shown). Moreover, 3-hydroxybutyrate did not modify cell death induction by activation of the mitochondrial apoptotic pathway via tumor necrosis factor-related apoptosis-inducing ligand (TRAIL; Figure 4C and data not shown), or inhibition of clonogenic cell growth by temozolomide, the adjuvant standard of care chemotherapeutic agent in glioma patient treatment (Figure 4D and data not shown). In all five glioma cell lines examined, neither synergistic nor antagonistic effects of 3-hydroxybutyrate were detected.

**Figure 5:**
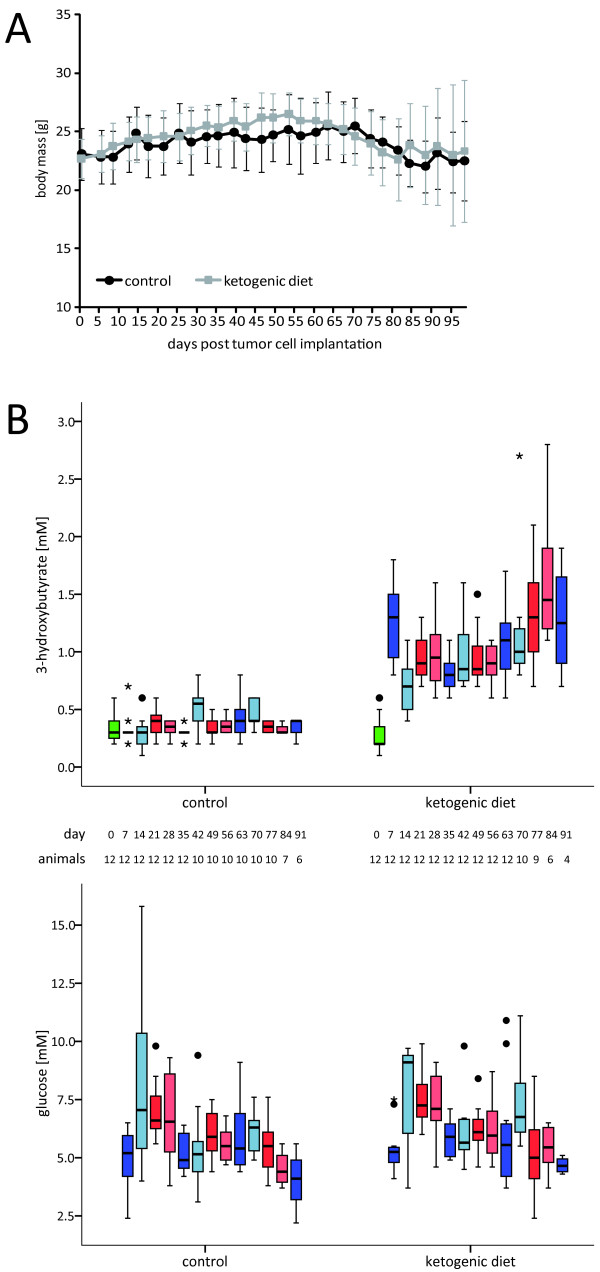
**Ketogenic diet induces ketosis but does not lower blood glucose levels**. LNT-229 cells were implanted into the right striatum of nude mice at day 0. Thereafter, animals were fed either the standard diet or the ketogenic diet. **(A) **Body weight was measured twice weekly and is presented as mean and standard deviation. **(B) **Blood levels of 3-hydroxybutyrate and glucose were determined on the day of tumor cell implantation (day 0) and every 7 days thereafter. 3-hydroxybutyrate values in mice fed the ketogenic diet were significantly higher than those in the control group (Bonferroni-adjusted p < 0.05 at all time points after diet change). By contrast, glucose levels did not differ significantly between diet groups. Boxplots depict the median, quartiles and extreme values. Upper and lower whiskers correspond to the highest and lowest values which are not greater than 1.5 times the interquartile range; •, cases with values between 1.5 and 3 times the interquartile range; *, cases with values more than 3 times the interquartile range.

**Figure 6:**
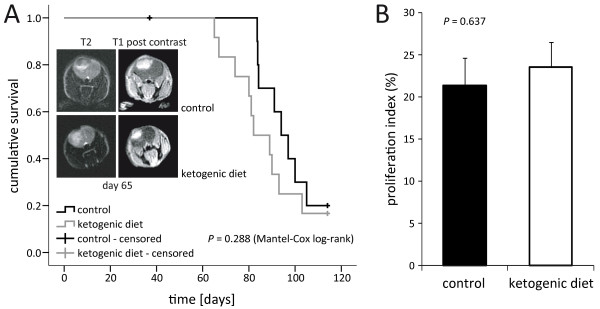
**Ketogenic diet fed *ad libitum *does not prolong survival in the LNT-229 xenograft model**. **(A) **Mice carrying LNT-229 xenografts were fed with ketogenic or control diet, observed in daily intervals and killed at the onset of neurological symptoms equal or worse than grade 2 (Kaplan-Meier survival estimate, Mantel-Cox log-rank p = 0.288). On days 37 and 65, magnetic resonance imaging of three randomly chosen animals from each group was performed. Representative images (day 65) are depicted. **(B) **Tumors of mice fed the ketogenic diet displayed proliferation (Ki-67 labeling) indices similar to those of control animals (mean and standard error of the mean, Student's t-test p = 0.637).

**Figure 7:**
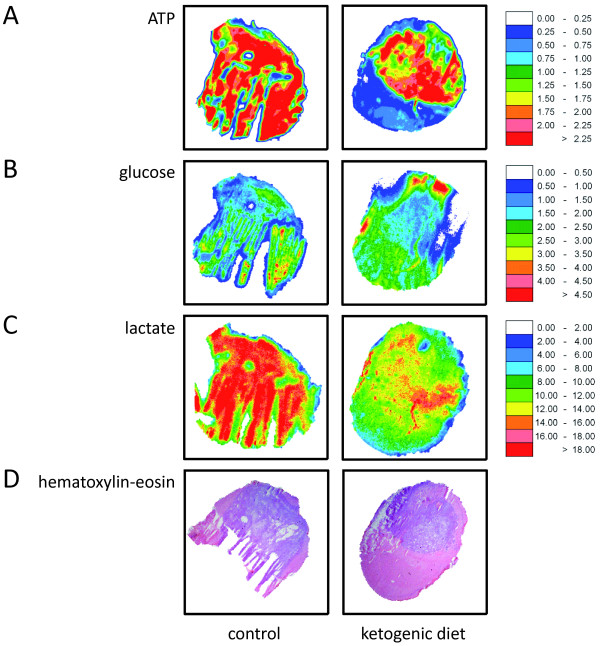
**Metabolic mapping does not reveal significant differences between the two diet groups**. Adjacent cryosections were used for hematoxylin-eosin staining **(D) **and for bioluminescence imaging of ATP **(A)**, glucose **(B) **and lactate **(C)**. Concentration distributions of these metabolites are color-coded [μmol/g].

*The ketogenic diet is well accepted and induces stable ketosis in vivo, but does not affect tumor growth or survival*. In a translational approach, the present study aimed to assess some characteristics of an unrestricted ketogenic diet similar to the one offered to patients in the pilot study carried out in our department (NCT00575146). Using the same ketogenic diet, Otto *et al*. had observed a delayed growth of subcutaneously implanted tumors of the gastric adenocarcinoma cell line 23132/87 [31]. After ruling out major tumor-supporting effects of ketone bodies *in vitro*, we performed an orthotopic xenograft mouse model experiment to assess whether this ketogenic diet might result in a tumor-selective energy deficit and thus reduce tumor growth *in vivo*. After orthotopic inoculation of LNT-229 glioma cells, mice were randomized into two non-calorie-restricted diet groups, a standard diet group and a ketogenic diet group. All animals readily accepted the unrestricted ketogenic diet. Similar to the findings of other studies [18,31], their body mass did not differ substantially from those of control animals (Figure 5A). Blood 3-hydroxybutyrate levels were continuously and significantly higher in mice fed the ketogenic diet than in animals on the control diet. By contrast, no relevant difference in blood glucose values was observed between the two groups (Figure 5B). Interindividual and intervisit blood glucose levels varied strongly. Similarly, serum concentrations of insulin-like growth factor 1 (IGF-1) were not significantly different between the two diet groups (Table 4). MRI analysis and observation for symptom-free survival did not reveal a difference between the two diet groups regarding tumor size and survival (Figure 6A). Histological analysis did not show differences in the proliferation rate (Figure 6B), nor in size or morphology of the tumors (data not shown). ATP, glucose or lactate concentrations in normal and tumor tissues did not differ significantly between the two diet groups (metabolite concentrations in tumor areas, mean and standard deviation, μmol/g, ATP: control, 1.9 ± 0.9, ketogenic diet, 1.9 ± 0.6, glucose: control, 1.6 ± 0.8, ketogenic diet, 2.0 ± 0.8, lactate: control, 19.8 ± 5.1, ketogenic diet, 20.9 ± 3.2; Figure 7). Consistent with the findings of other studies [43,44] but not reaching statistical significance, bioluminescence imaging data suggested lower glucose and higher ATP and lactate concentrations in tumors than in corresponding normal tissues (tumor-to-normal tissue ratio, mean and standard deviation, ATP: 1.59 ± 0.91, glucose: 0.88 ± 0.12, lactate: 1.29 ± 0.55).

**Table 4:** Murine serum IGF-1 concentrations during the ketogenic diet

	days post tumor cell implantation							
	**14**		**24**		**38**		**65-115**	

	**control**	**KD**	**control**	**KD**	**control**	**KD**	**control**	**KD**

animals	4	4	3	3	3	3	10	9

**IGF-1 [ng/mL]**	245.0 ± 50.3	310.3 ± 36.8	310.0 ± 28.9	417.5 ± 106.1	312.7 ± 84.9	301.9 ± 36.5	242.8 ± 53.2	292.2 ± 58.1

After LNT-229 tumor cell implantation at day 0, animals were randomized into two diet groups, a standard diet versus a ketogenic diet (day 1). On days 14, 24 and 38 as well as at the end of the experiment (days 65-115), serum was obtained by centrifugation of whole blood at 2000 g for 10 min and subsequently stored at -80°C. Murine IGF-1 serum levels determined by immunoassay were not significantly different between diet groups (KD, ketogenic diet; mean and standard deviation).

## Discussion

Strategies specifically targeting the altered metabolic pathways of tumors are increasingly receiving attention [45,46]. In contrast to new chemotherapeutic approaches, altering diet is perceived by many as an easy option to delay tumor progression with few side effects. To the best of our knowledge, however, no randomized clinical trials have been initiated on ketogenic diets for therapy in any tumor type. Concerning nutrition, patients see themselves faced with incomplete information and contradictory, sometimes extreme recommendations. Given the principle of *primum nil nocere*, we therefore considered it reasonable and necessary to preclinically evaluate effects of dietary changes just like those of other anticancer drugs.

Cerebral ketone body metabolism depends on (i) concentrations in blood, (ii) the transport across the blood-brain barrier and into cells and (iii) the activity of ketone body metabolizing enzymes [6]. Blood concentration is considered the most important factor affecting the rate of cerebral ketone body metabolism. As the blood-brain barrier is relatively impermeable to most hydrophilic substances, transporters for short-chain monocarboxylic acids (MCTs, SLC16 family [47]), such as ketone bodies and lactate, are needed and govern access of acetoacetate and 3-hydroxybutyrate to central nervous system tissues [48]. Ketone body entry into brain cells occurs by diffusion (at a moderately high rate) and carrier-mediated processes (MCTs; probably less regulated). Finally, ketone body metabolism depends on the activities of the relevant enzymes, but due to the limited number of studies [49], sufficient information concerning those enzymes in humans is not available.

We here demonstrate a deficient utilization of the major ketone body 3-hydroxybutyrate by human malignant glioma cells. 3-hydroxybutyrate did not alter cell density or proliferation and could not protect the human glioma cell lines examined against glucose deprivation. By contrast, this ketone body conferred protection from glucose withdrawal-induced cell death in primary rat hippocampal neurons (Figure 2). A recent study using organotypic rat hippocampal slice cultures supports this observation [50], being in accordance with the capability of rats to metabolize ketone bodies [51,52]. Neuroprotective effects of 3-hydroxybutyrate on primary spinal cord neurons from SODI-G93A mice and on SH-SY5Y human dopaminergic neuroblastoma cells exposed to the complex I inhibitor rotenone have also been described [53,54]. In our experiments, no protective effect of 3-hydroxybutyrate on glioma cell death induced either by rotenone or by the glycolysis inhibitor 3-bromopyruvate was detectable (Figure 4A-B). Hence, in those cell lines, this ketone body could not compensate for energy depletion induced by disturbance of the mitochondrial respiratory chain or glycolysis. Further, we did not observe proapoptotic effects of ketone bodies in glucose-free medium as did Skinner *et al*. using the human neuroblastoma cell line SK-N-AS [55], indicating that this effect might be cell type specific. An increase in HIF-1α protein levels caused by accumulation of succinate and inhibition of PHD enzymes (see Figure 1A) has been described in diet-induced ketotic as well as in 3-hydroxybutyrate-infused rat brain [56] and would probably be a rather unwanted effect regarding tumor treatment [57,58]. We therefore evaluated expression, transcriptional activity and target gene modulation of HIF-1α. However, neither was affected by 3-hydroxybutyrate (Figure 3 B-C; Additional file 2, Figure S2), suggesting that this ketone body is not metabolized in the glioma cell lines examined. Furthermore, 3-hydroxybutyrate did not influence the migratory behavior of the glioma cell lines (Table 3). By contrast, it has been observed that 3-hydroxybutyrate like lactate may function as a chemoattractant, stimulating the migration of MDA-MB-231 human breast cancer cells *in vitro *[59].

Together, our *in vitro *experiments did not indicate any ketone body metabolizing activity of the glioma cell lines examined, a finding which contrasts the protective effect of 3-hydroxybutyrate on rat hippocampal neurons. The observed phenotype is probably not caused by deficient expression of monocarboxylic acid transporters (Additional file 2, Figure S2, [60]) or ketone body metabolizing enzymes (Figure 1B). Both neurons and astrocytes are in principle capable of using ketone bodies as metabolic fuels [61-63]. In tumors, citric acid cycle metabolism is supposed to be intact and important for their ability to synthesize substrates for membranes, nucleic acids and proteins [64,65]. In our experiments, the accumulation of HIF-1α observed after treatment with 3NPA also indicates an intact citric acid cycle in the glioma cell lines (Figure 3D). Other studies on malignant gliomas demonstrated reductions in electron transport chain activities [11], structural defects [10,66] and DNA mutations [67] in mitochondria. Nonetheless, glioma cells have been shown to exhibit [10] and be able to modulate respiratory activity [68]. Therefore the basic requirements for energy yield from ketone bodies (enzyme equipment, intact citric acid cycle and respiratory chain) should be met. Alternatively, the 3-hydroxybutyrate offered could have been used primarily for the synthesis of lipids, but in this case a decrease in supernatant concentrations and perhaps an effect on proliferation would have been expected. However, the pure presence of a protein does not imply its proper function and we didn't analyze activities of ketone body metabolizing enzymes in the present study. In an analysis of various tumors and tissues of the nervous system, OXCT1 enzyme activity was found to be lower in glial tumors compared to normal human brain [15]. So enzyme activity could be the factor limiting ketone body metabolism [6,18]. Taken together, we observed a lack of capability to degrade ketone bodies in the glioma cell lines. The deficiency in metabolizing ketone bodies might indeed represent a characteristic of malignant transformation, but the underlying defect remains unclear.

A reduction of tumor growth under conditions of caloric restriction and/or weight loss has repeatedly been shown in glioma models [17,18]. However, we rarely observe chemotherapy-associated (unintended) weight loss in brain tumor patients and a non-calorie-restricted ketogenic diet might be more easily realized than calorie restriction. We therefore performed an *in vivo *experiment using an unrestricted ketogenic diet. The diet was well accepted, and no significant differences developed in body weight between the two diet groups (Figure 5A). Further, glucose concentrations and IGF-1 levels did not differ substantially between the groups (Figure 5B,Table 4). These results are consistent with prior analyses showing no decline in blood glucose concentrations when a ketogenic diet was administered in unrestricted amounts [18,31,69]. According to other studies [17,69,70], a reduction in circulating IGF-1 levels would have been expected only under conditions of caloric restriction. Thus, the stable values of body mass, glucose and IGF-1 help to distinguish between possible starvation-associated effects and other metabolism-specific effects of the ketogenic diet. Finally, the ketogenic diet alone did not influence tumor growth in the glioma model used (Figure 6A); tumor histopathology and metabolic mapping revealed no differences between mice fed the ketogenic diet and control animals (Figure 7). Although these results are consistent with those obtained in other syngeneic (CT-2A) and xenogeneic (U87MG) glioma mouse models [18], they contrast with the findings of Otto *et al*. where a diet identical to the one used in our *in vivo *experiment significantly decreased the growth of subcutaneously implanted tumors of the gastric adenocarcinoma cell line 23132/87 [31]. The different results of the latter and our study most likely reflect intrinsic properties of the cell lines used. In contrast to the gastric adenocarcinoma cell line, LNT-229 human glioma cells usually form quite homogeneous non-necrotic tumors. Necrotic tumors might be more susceptible to dietary restriction, as the necrotic areas in those tumors reflect the already limited supply of nutrients and oxygen. Since hypoxic tumor cells particularly depend on glucose availability [37], limiting carbohydrates might be effective in these tumors. The downregulation of ketone body metabolizing enzymes under hypoxic conditions observed in our cell lines (Figure 3A) suggests an additional disadvantage of this hypoxic tumor fraction. Another possible explanation may be a differential energy supply of subcutaneous and intracranial tumors. The mouse brain cells could have adapted to metabolize ketone bodies, leaving enough glucose to meet the xenografts' energy requirements. Using a different unrestricted ketogenic diet and the GL261 syngeneic intracranial glioma mouse model, Stafford *et al*. found a reduced rate of tumor growth and prolonged survival [71]. Likewise, GL261 tumors display necrotic zones [72]. Obviously, the impact of ketosis and hence the results of such studies depend on the model applied.

## Conclusion

In summary, our results suggest a deficiency of glioma cell lines to metabolize ketone bodies *in vitro*, supporting the possibility of targeting tumor energy metabolism by a ketogenic diet. However, an unrestricted ketogenic diet was not effective as a monotherapy in the xenograft model applied. A combination of a ketogenic diet with strategies inhibiting glycolysis or interfering with the tumor's energy supply, such as vascular disrupting or antiangiogenic agents [73], might result in synergistic antitumor effects and is worth further investigation.

## Abbreviations

3OHB: 3-hydroxybutyrate; AACS: acetoacetyl-CoA synthetase; AcAc: acetoacetate; ACAT1: acetyl-CoA acetyltransferase; BDH: 3-hydroxybutyrate dehydrogenase; GLUT1: glucose transporter 1; HIF-1α: hypoxia-inducible factor-1α; HRE: hypoxia responsive element; IGF-1: insulin-like growth factor 1; MCT4: monocarboxylic acid transporter 4; OXCT1: 3-oxoacid-CoA transferase 1; TRAIL: tumor necrosis factor-related apoptosis-inducing ligand; VEGF: vascular endothelial growth factor.

## Competing interests

The authors declare that they have no competing interests.

## Authors' contributions

GDM participated in the design of the study, carried out *in vitro *and *in vivo *experiments and drafted the manuscript. DPB participated in RNA and animal experiments, OB in animal experiments. PNH performed the histological analysis. EH conducted magnetic resonance imaging. SW and WMK realized metabolic mapping with quantitative bioluminescence and single photon imaging. JPS and JR conceived of the study and helped to compose the manuscript. All authors read and approved the final manuscript.

## Pre-publication history

The pre-publication history for this paper can be accessed here:

http://www.biomedcentral.com/1471-2407/11/315/prepub

## Supplementary Material

Additional file 1 **Figure S1**. The presence of 3-hydroxybutyrate does not modify glucose consumption or lactate generation of the glioma cell lines. U87MG, U251MG, LNT-229, T98G and A172 cells were cultured in medium containing 5 mM glucose and 5 mM 3-hydroxybutyrate (3OHB). Glucose and lactate concentrations of cell culture supernatants were analyzed on days 1, 2, 3, 4, 5 and 6. Click here for file

Additional file 2 **Figure S2**. Expression of HIF-1α target genes is not modulated by 3-hydroxybutyrate. LNT-229 cells were either untreated or treated with 3-hydroxybutyrate for 24 h at normoxia or hypoxia, and the expression of the HIF-1α target genes *GLUT1, VEGF *or *MCT4 *was analyzed by real-time quantitative PCR (fold change in gene expression normalized to the internal control 18S rRNA; mean and standard deviation). Click here for file
