# Supplementary figures and images for: Differential utilization of ketone bodies by neurons and glioma cell lines: a rationale for ketogenic diet as experimental glioma therapy
Source: BMC Cancer. 2011 Jul 26;11:315. doi: 10.1186/1471-2407-11-315 (PMC3199865; doi:10.1186/1471-2407-11-315)

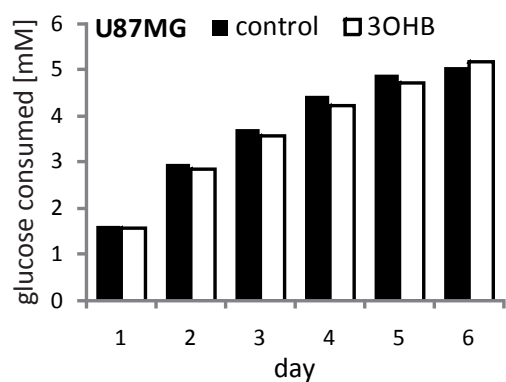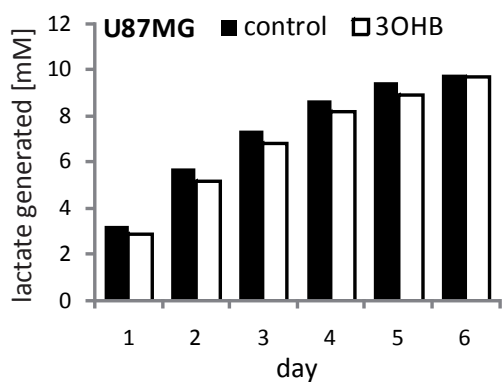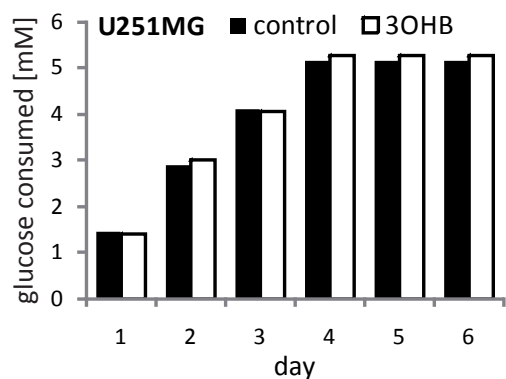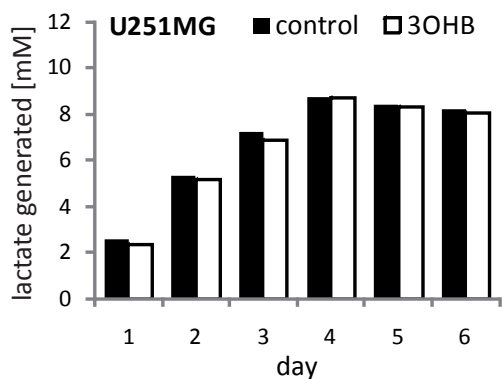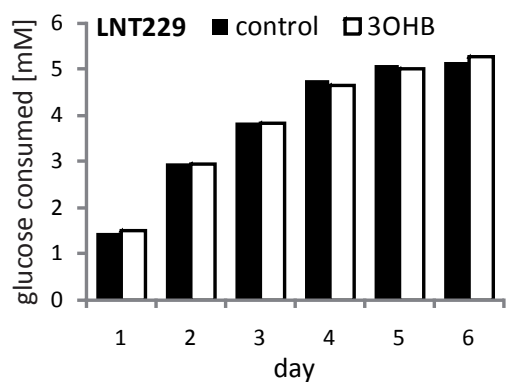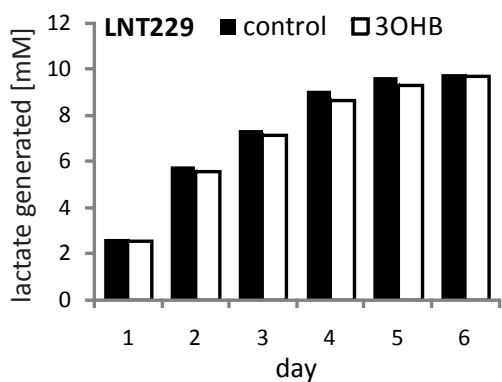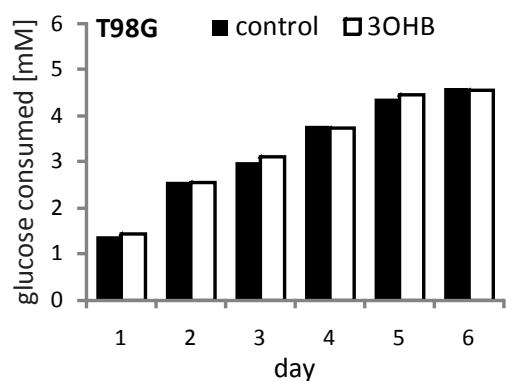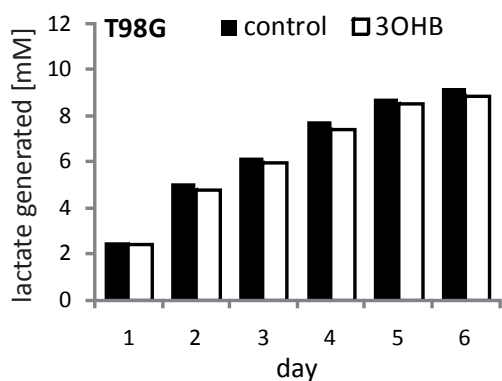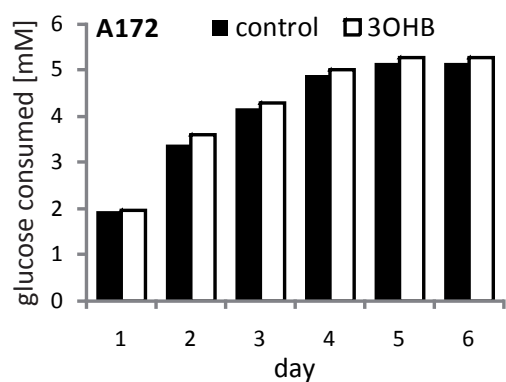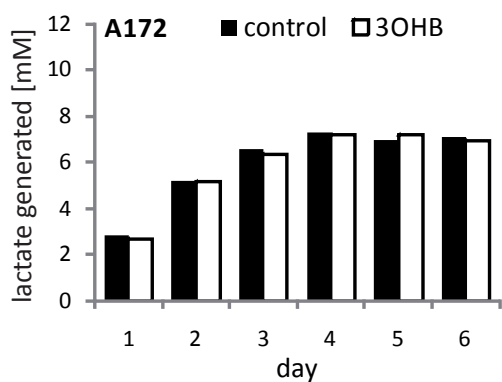

Supplement: Additional file 1 — Figure S1. The presence of 3-hydroxybutyrate does not modify glucose consumption or lactate generation of the glioma cell lines. U87MG, U251MG, LNT-229, T98G and A172 cells were cultured in medium containing 5 mM glucose and 5 mM 3-hydroxybutyrate (3OHB). Glucose and lactate concentrations of cell culture supernatants were analyzed on days 1, 2, 3, 4, 5 and 6. [file 1471-2407-11-315-S1.PDF]

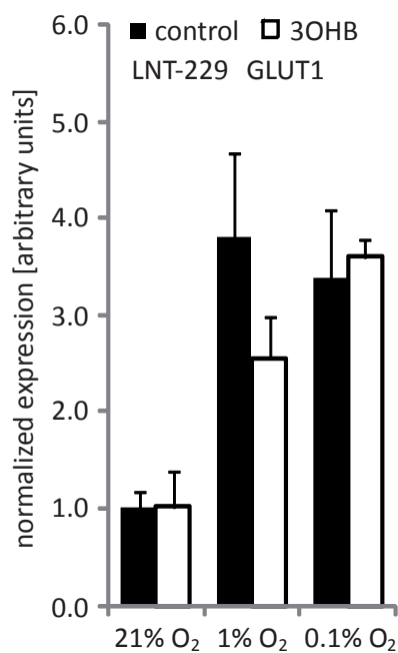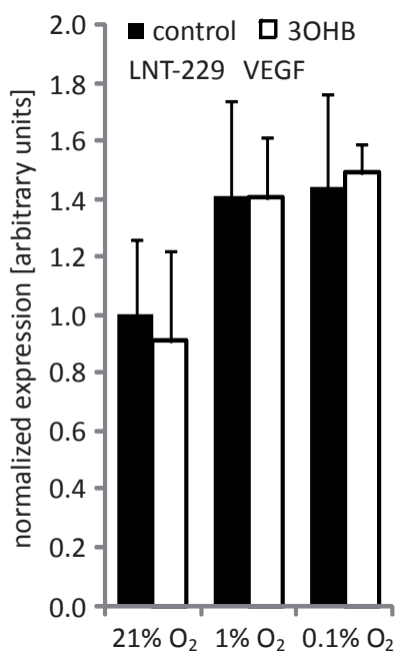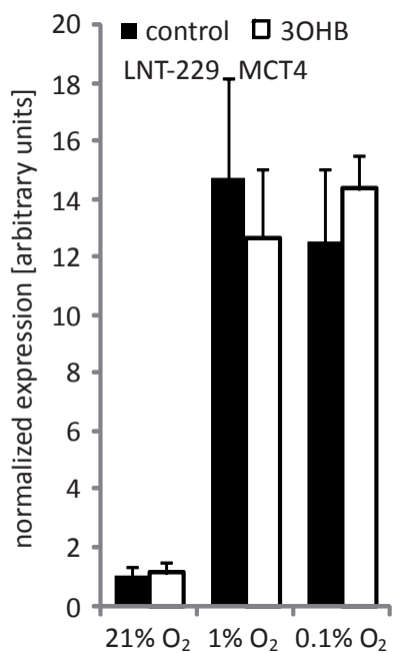

Supplement: Additional file 2 — Figure S2. Expression of HIF-1α target genes is not modulated by 3-hydroxybutyrate. LNT-229 cells were either untreated or treated with 3-hydroxybutyrate for 24 h at normoxia or hypoxia, and the expression of the HIF-1α target genes GLUT1, VEGF or MCT4 was analyzed by real-time quantitative PCR (fold change in gene expression normalized to the internal control 18S rRNA; mean and standard deviation). [file 1471-2407-11-315-S2.PDF]
